# *MMX-I*: data-processing software for multimodal X-ray imaging and tomography

**DOI:** 10.1107/S1600577516003052

**Published:** 2016-04-12

**Authors:** Antoine Bergamaschi, Kadda Medjoubi, Cédric Messaoudi, Sergio Marco, Andrea Somogyi

**Affiliations:** a Synchrotron SOLEIL, BP 48, Saint-Aubin, 91192 Gif sur Yvette, France; b UMR 9187, Université Paris-Saclay, CNRS, Université Paris-Saclay, F-91405 Orsay, France; c U1196, Institut Curie, INSERM, PSL Reseach University, F-91405 Orsay, France

**Keywords:** scanning multimodal X-ray imaging, scanning X-ray tomography, tomographic reconstruction, phase retrieval, scanning multimodal X-ray imaging, scanning X-ray tomography, tomographic reconstruction, phase retrieval

## Abstract

A new multi-platform freeware has been developed for the processing and reconstruction of scanning multi-technique X-ray imaging and tomography datasets. The software platform aims to treat different scanning imaging techniques: X-ray fluorescence, phase, absorption and dark field and any of their combinations, thus providing an easy-to-use data processing tool for the X-ray imaging user community. A dedicated data input stream copes with the input and management of large datasets (several hundred GB) collected during a typical multi-technique fast scan at the Nanoscopium beamline and even on a standard PC. To the authors’ knowledge, this is the first software tool that aims at treating all of the modalities of scanning multi-technique imaging and tomography experiments.

## Abstract 2

The *MMX-I* open-source software has been developed for processing and reconstruction of large multimodal X-ray imaging and tomography datasets. The recent version of *MMX-I* is optimized for scanning X-ray fluorescence, phase-, absorption- and dark-field contrast techniques. This, together with its implementation in Java, makes *MMX-I* a versatile and friendly user tool for X-ray imaging.

## 1. Introduction   

**Figure 1:**
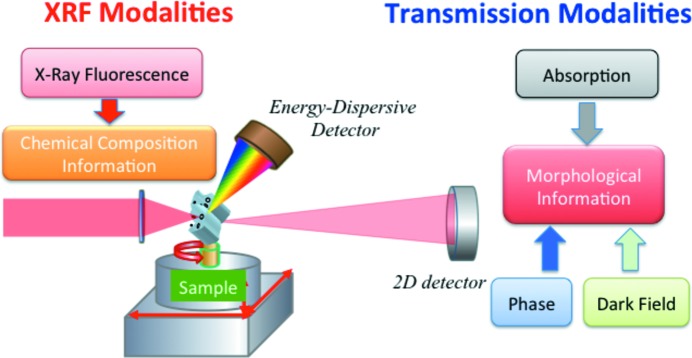
Principle of the scanning nanoprobe station. The sample is raster scanned in the focused beam while recording simultaneously the XRF spectra by energy-dispersive detectors and the transmitted beam by a fast and sensitive 2D detector. The resulting data matrix includes the XRF spectra, the 2D magnified transmission images, the values of the intensity, and beam position monitors and sample scan positions.

The Nanoscopium beamline of Synchrotron SOLEIL (Somogyi *et al.*, 2015 ▸) is dedicated to scanning multi-technique X-ray imaging in the 5–20 keV energy range. It aims to offer two-dimensional/three-dimensional (2D/3D) quantitative information on the elemental composition and electronic density of samples with high spatial resolution (down to 50 nm in 3D) and analytical sensitivity, *i.e.* trace, sub-parts-per-million (sub-p.p.m.), detection limit. The beamline is especially well suited for hierarchical length-scale studies of highly heterogeneous samples providing simultaneous morphological, elemental and chemical information at multiple length scales. Indeed, the scanning range can be scaled from millimetres to micrometres with spatial resolution down to 50 nm. The main scientific fields of application at the beamline are biology, life sciences, geo-biology and environmental sciences. Distribution of biologically important transition metals in organic tissues and cells (Colvin *et al.*, 2015 ▸), elemental composition and morphology of geological and paleo-geological samples (Sforna *et al.*, 2014 ▸), metal uptake and sequestration mechanisms in plants are some representative examples of research which can be performed at the Nano­scopium beamline. The multi-technique ‘FLYSCAN’ data acquisition scheme developed at Synchrotron SOLEIL (Medjoubi *et al.*, 2013*b* ▸; Leclercq *et al.*, 2015 ▸), based on the specific technical features of Nanoscopium, is crucial for such multi-length scale scanning imaging. It permits scanning X-ray fluorescence spectrometry combined with absorption, differential phase contrast and dark-field imaging to be performed with down to millisecond dwell time per pixel. As such, large-field-of-view measurements become feasible in a few hours of measurement time. The principle of the scanning nanoprobe station is presented in Fig. 1 ▸. During a usual experiment a large overview map of 2000 × 2000 pixels (∼2 mm × 2 mm) is recorded with millisecond dwell time per pixel. This results in a half-terabyte dataset in about 1 h. The large overview mapping is followed by a more resolved, but slower, scan performed on a ≤100–200 µm^2^ sized region. This produces a similar amount of data in 1–2 h. Obviously, on-line and off-line data processing is a crucial part of the success of the experiments. However, the treatment of such large datasets, regularly generated during synchrotron user experiments, still represents a challenge. These large amounts of raw data have to be imported, reduced, corrected and pre-processed within the time frame of the experiment for on-site data viewing. Moreover, it must also be feasible to perform these data treatment steps on a standard PC by users for thorough post-experimental data processing in their own home institutes, where they often do not possess a high-performance workstation. Such data-reconstruction software and algorithms must be easy to be used as well by users inexperienced in image processing. In order to cope with these challenges, we have developed a dedicated data processing tool at the Nanoscopium beamline, which will be proposed as freeware for the scientific community.

One of the main requirements for such a software tool, both for 2D imaging and tomography, is to extract the physical properties from the samples encoded in the raw data [recorded at SOLEIL in a single NeXus file (Poirier *et al.*, 2009 ▸)]. These properties are transformed into images of phase, scattering contrast, absorption contrast and elemental distribution maps. Moreover, calibrations and corrections for detector imperfections (*e.g.* hot pixels of the 2D detector) and for eventual positioning irregularities of the sample stage, have to be performed in a transparent manner. Furthermore, due to the large variety of samples, the sampling strategy and the recorded signals can be very different from one experiment to another (*e.g.* intensity, background). As such, several tomographic reconstruction algorithms and phase-retrieval methods, tailored to the different measurement strategies and imaging contrasts, must be proposed.

Many open-source software or frameworks exist for transmission tomographic reconstruction or phase retrieval, such as *TomoPy* (Gürsoy *et al.*, 2014 ▸), *TomoJ* (Messaoudii *et al.*, 2007 ▸), *AnkaPhase* (Weitkamp *et al.*, 2011 ▸) and *PITRE* (Chen *et al.*, 2012 ▸) (a non-exhaustive list). However, they are mostly oriented for full-field imaging and not for scanning imaging. To our knowledge, the open freewares mostly dedicated for scanning X-ray fluorescence (XRF) and X-ray absorption near-edge structure (XANES) applications are *PyMCA* (Solé *et al.*, 2007 ▸), *Mantis* (Lerotic *et al.*, 2014 ▸), *MAPS* (Vogt, 2003 ▸), *TXM-Wizard* (Liu *et al.*, 2012 ▸) and *Axis2000* (aXis2000, 1997 ▸).

This made it necessary to develop the open-source *MMX-I* software for the off-line processing of multimodal scanning X-ray imaging and tomography datasets. Taking into account the already existing widely used XRF spectrum-fitting software (*e.g.* *PyMCA*), we did not intend to develop such fitting tools for *MMX-I*. Instead, it is possible to import *PyMCA* results, namely elemental intensities obtained by fitting each pixel spectra, into *MMX-I* for further processing.

*MMX-I* has been designed following a modular architecture, based on the model view controller (MVC) pattern (Reenskaug, 1979 ▸). This allows enlarging its application even beyond scanning imaging by including the processing of any other imaging datasets requiring tomographic or phase reconstruction.

## 2. *MMX-I* for data reduction and processing   

### 2.1. Software platform and workflow   

The *MMX-I* project aims to offer both expert users and beginners the possibility of processing and analysing raw data, either on-site or off-site, for each scanning imaging technique included in the software and any of their combinations. Therefore we have developed a multi-platform (Mac, Windows and Linux 64-bit) data processing tool, which is easy to install, comprehensive, intuitive and user friendly.

As software developments based on scripting languages, such as Matlab or IDL, require the installation of rather expensive software packages, we preferred to develop *MMX-I* in Java. Java has a good synergy with the widely used (by different scientific communities) image processing software *ImageJ* (Saalfeld *et al.*, 2012 ▸; Schneider *et al.*, 2012 ▸; Rizk *et al.*, 2014 ▸). Several advantages have been considered with this choice: (i) Java code can run on all Java-supporting platforms without the need of recompilation; (ii) the software tool can be installed with a single executable file, which can even include the appropriate Java version; (iii) the application can be launched from a USB stick and, as such, is easily delivered to the users; (iv) Java includes a large number of scientific class libraries, which allows the easy development of numerical calculations adapted to our needs; (v) *ImageJ* libraries provide the basic image visualization and processing tools.

**Figure 2:**
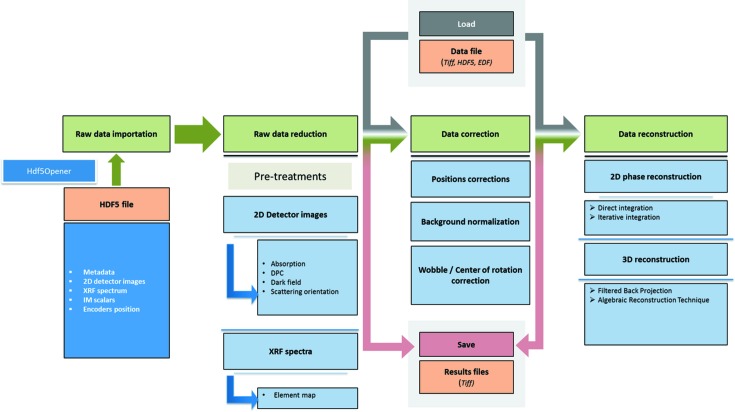
*MMX-I* workflow. Raw data are imported from an HDF5 file *via* Hdf5Opener and then reduced to absorption, dark-field, phase-contrast (DPC) and elemental maps. After this step data can be exported as tiff files or existing maps can be imported. This is followed by data correction and then data reconstruction.

The *MMX-I* workflow is presented in Fig. 2 ▸. The data processing chain is divided into four modules: data importation, reduction, correction and reconstruction. Each module is detailed in the following sections.

### 2.2. Implemented modules   

#### 2.2.1. Data importation   

**Figure 3:**
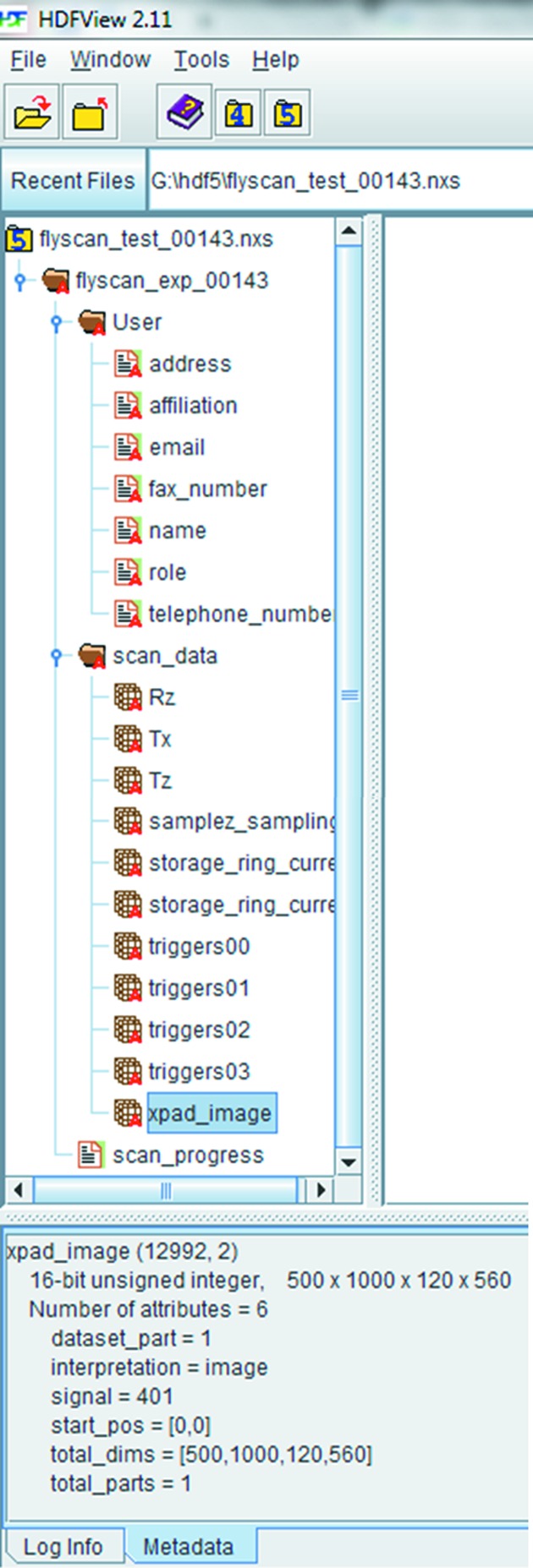
File tree of a single NeXus file corresponding to a 2D multi-technique scan. The images of the bi-dimensional detector are contained in a 4D matrix (each pixel of the 2D data map contains a 2D detector image), XRF spectra are in a 3D matrix, the positions and intensity values are in a 2D matrix.

At SOLEIL, raw data including metadata (such as scan parameters, sample identifier, beamline parameters and timestamp), obtained during an experiment, are recorded in an HDF5 (The HDF Group, 2015*a* ▸) file following the NeXus convention (Könnecke *et al.*, 2015 ▸). Fig. 3 ▸ presents a file tree of a single NeXus file of data taken during a multi-technique X-ray 2D scanning experiment at Nanoscopium. Being an HDF5 file it is opened using the *HDFView* software (The HDF Group, 2015*b* ▸). The hierarchical HDF5 format facilitates data processing and interpretation, because the data from each subsystem (sensors, motors, *etc.*) are recorded in a single HDF5 file following the order of the acquisition process. The HDF5 is a binary file indexed in B-tree (tree data structure). In addition to being self-describing, the HDF5 format is quasi-unlimited in size (*i.e.* limited by the file system). HDF5 is well supported and continuously improved by the HDF Group in order to stay at the cutting edge of innovation.

**Figure 4:**
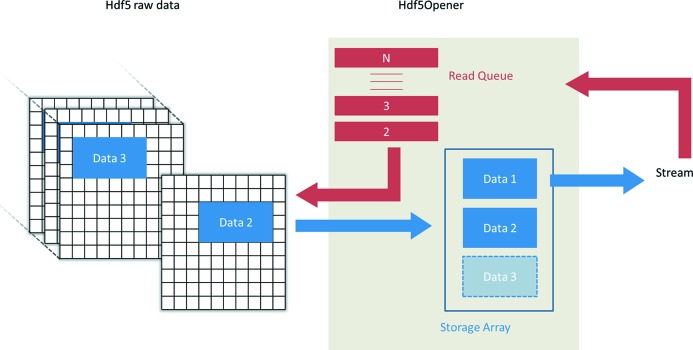
Scheme of the Hdf5Opener streaming process. The ‘Stream’ object passes to the Hdf5Opener (red arrow) to the read queue which consists of a list of read requests. Hdf5Opener reads sequentially, in ‘continuous’ mode, each read request and stores the result in the storage array (blue arrow).

Reading and processing large HDF5 files of 0.5–1 TB volumes, produced regularly by Flyscan at Nanoscopium, requires high computing performances, which is not available by standard PC or laptop computers. As such, the optimization of the computing resources was a crucial part of the *MMX-I* project in order to cope with the handling of big data files even by standard user-PCs. For this purpose, a dedicated readout API, named Hdf5Opener, has been developed. Indeed, reducing data takes only a small fraction of the computation power compared with file readout. The bottlenecks of the reduction procedure are the memory available for the readout of the complete dataset contained in the file and the time necessary for data transfer from the disk to the memory. In order to address these problems, Hdf5Opener owns its own dedicated memory and execution thread, which allows working in parallel with the other processing threads of *MMX-I*. The strategy is to partially read the Hdf5 file and to separate data reading and processing. In detail, as shown in Fig. 4 ▸, a stream object sends to the API a list of read requests, each containing the selection of the partial dataset to be sequentially read out. After the readout of the first request of the list, the block of data is stored in the storage array and queued up to be processed. The operation is performed continuously for all elements of the list, according to the available memory size. Once one of the parallel-processing tasks on a given block of data is terminated, the corresponding memory space in the storage array is released. By default, the size of the block of data is set to be equal to the size of the first dimension of the scan. As an example, for a 2D scan, the block of data will contain the elements of one line. This strategy is detailed in §2.2.2.

The data contained in each block may not be necessarily adjacent in the pixel map. Indeed, for rapid overview of very large datasets, the API can read each second pixel of the map in all dimensions. This mode is called ‘low resolution’ in *MMX-I* and is described below. Moreover, it can be decided not to process parts of the dataset which are considered to be uninteresting (region outside of the sample or corresponding to beam loss *etc*.).

#### 2.2.2. Data reduction   

Data reduction consists of processing X-ray transmission and XRF raw data to obtain: (i) images from the transmitted signal; (ii) horizontal and vertical differential phase contrast images; (iii) dark-field images; (iv) the orientation of the scattering (to be implemented in the near future); (v) elemental distribution maps.

Previous to data reduction, raw data have to be pre-treated. For instance, the signal type (absorption, phase, dark field and fluorescence) contained in the treated data matrix has to be identified, denoised and calibrated.

**Figure 5:**
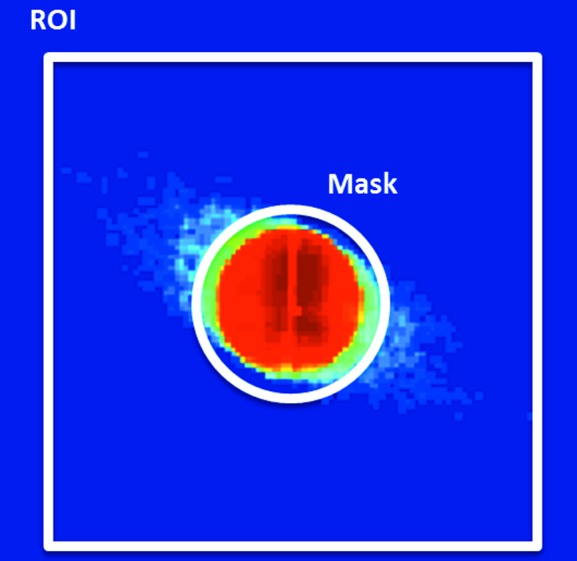
Transmitted X-ray beam recorded by the XPAD 2D photon-counting detector. The image is displayed on a log scale. Pixels used for data reduction are inside the default ROI marked with a white square. The white circle represents the outer limit of the default ‘illumination mask’ used for determining the pixels belonging to the dark field.

*Pre-treatment.* Usually, the largest part of the total data volume is represented by the transmission images. In order to save computing resources and time, only those image-fractions [image region of interest (ROI)] of the detected raw transmission images will be read by Hdf5Opener, which contains ‘significant’ information for calculating the transmission modalities. To this end, when the raw data file is loaded for the first time, *MMX-I* will automatically read 10% of the whole transmission dataset, uniformly distributed in the coordinates of the scan. From this step, the ROI containing the whole transmitted and scattered beam is determined (see Fig. 5 ▸). In a second step, a mask of the illumination pattern is defined, which is especially important for the calculation of the dark-field images as described later. The ROI images are thresholded with a minimal value, transformed to binary images and then summed. The resulting ‘illumination mask’ represents for each pixel the number of times the pixel was illuminated by X-rays (above the threshold value) during the whole scan. To obtain a reasonable transmitted beam shape we only consider the pixels which are illuminated within the 95–100% range of the maximum value of the illumination mask (see Fig. 5 ▸). This ROI and illumination mask are then proposed to the user as the default ones (see §2.5). The default ROI and illumination mask can be customized by the user.

Hot pixels are removed from each 2D detector image before further data processing. A dedicated function has been developed in order to identify and remove those pixels, which are significantly different from their neighbours. For this, a local histogram statistics algorithm based on the variance value of each pixel in its neighbourhood (Gonzalez & Woods, 2006 ▸) is used. The available default parameters such as the convolution kernel, used to compute the variance, or the histogram threshold can be modified by the user. The positions of the reliably identified hot pixels are then recorded in a look-up table. Then, they are either replaced by the average intensity value of the neighbouring pixels or not considered at all in the reduction processes.

XRF is an analytical method for determining the chemical composition of the specimen. When a sample is exposed to an X-ray beam with sufficient energy, characteristic X-ray fluorescence radiation will be emitted with discrete energies that are specific of the excited elements. By measuring their energies and intensities, it is possible to determine the concentration of the measured elements. This information for a given element is obtained by measuring, for each pixel of the map, the area of the corresponding peak. Energy calibration of the XRF spectra is a crucial step for the identification of the elements contained in the sample and the reconstruction of the elements maps. By default, *MMX-I* calculates the sum spectra separately for each X-ray fluorescence detector. The energy calibration of the spectrum is performed in the standard way (Grieken & Markowicz, 2001 ▸), by selecting at least two known X-ray lines. Then, the user can interactively select ROIs, containing the characteristic X-ray peaks of interest, which will be used during the further data reduction process. XRF spectrum deconvolution and background subtraction have not been integrated in *MMX-I*, since the strategy was not to re-create existing functions, which have been optimized and widely used in excellent software for several years, *e.g.* *PyMCA* (Solé *et al.*, 2007 ▸). Instead, *MMX-I* provides the possibility to export sum spectra in ‘*mca*’ file format in order to be processed by *PyMCA*. The resulting ‘fit’ file can be imported into *MMX-I* in order to create ROI images. An additional option allows fitted elemental maps obtained by *PyMCA* to be re-injected into *MMX-I* for further processing, *e.g.* for tomographic reconstruction.

*Data reduction step.* The data reduction step is performed by default for all the pixels of the scan. The transmission modalities are determined from the images of the transmitted beam (see Fig. 5 ▸). The transmission contrast image is calculated by the numerical summation of the pixels within the beam ROI (see the *Pre-treatment* section above) giving access to absorption contrast typical of conventional radiography.

The differential phase contrast (DPC) is proportional to the refraction angle (Mukaide *et al.*, 2009 ▸) as described in the following formulae,
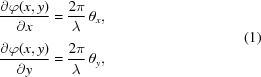
where φ is the phase, 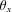 and 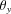 are the respective refraction angles along the horizontal and vertical directions, and λ is the wavelength. The refractions angles are determined by calculating the centroid of the transmitted beam, inside the ROI, along the horizontal and vertical directions. DPC images are used for phase map reconstructions (described below). It has to be noted that the scanning DPC technique allows the phase to be measured beyond 2π and therefore does not suffer from any phase-wrapping effects (de Jonge *et al.*, 2008 ▸).

The dark-field data represent the intensity of the scattering signal. Its amplitude is linked to the density fluctuation on a length-scale smaller than the illuminated area (Menzel *et al.*, 2010 ▸) and is evaluated by integrating the scattered intensity outside of the transmitted beam, *i.e.* outside the illumination mask shown in Fig. 5 ▸. The anisotropy of the scattering can also be exploited and provides contrast information on the orientation of structures in the sample. Orientation may be retrieved by dividing each integrated area of a large annulus ROI into smaller parts, spread over 360°, and then computing the angle by fitting the intensity of each part with a cosine function (Bunk *et al.*, 2009 ▸).

### 2.3. Data correction   

In order to improve the quality of the reduced data images, an additional correction step is performed. This allows users to correct the effects of the beam intensity variation, the translation stage positioning errors, the image background variation and, for tomographic acquisition, the slight misalignment and eccentricity (called wobble) of the rotation axis. Correction of the reduced images is a crucial step in order to obtain reconstructed images with a high degree of fidelity. It is the reason why this image processing has been carefully developed in *MMX-I*.

In order to correct for the variation of the incoming beam flux, an intensity monitor is inserted upstream of the sample. As such, the transmission, XRF and dark-field modalities are normalized with the incoming beam intensity measured in each pixel.

In fast-scanning multi-technique nanoprobe methods, the sample is scanned in continuous motion along one dimension while the detectors (intensity monitor, 2D detector and XRF detectors) and the encoder positions are recorded for each pixel/voxel. The simultaneous data acquisition of each device is started by an external trigger signal. In the FLYSCAN architecture, the triggers are provided by a periodic TTL pulse generator. Due to motor acceleration/deceleration, motion speed stability and backlash, the pixel size of the map will not be equivalent in the whole scan. This inhomogeneity can strongly restrain the use of algorithms requiring data continuity. Therefore, each image has to be rescaled by the recorded encoder positions. *MMX-I* includes an algorithm to correct for such imperfections by positioning each measured pixel in a new perfect virtual grid. The virtual grid is created based on the measured motor position of each pixel. This grid is first reshaped in order to have pixels of the same size only. The new pixel size is defined as having the mean height and width of the measured pixels. The value of each measured pixel is then re-assigned in the virtual grid. Pixels from the virtual grid may contain the value of several measured pixels or none. To avoid these brutal discontinuities the virtual grid is finally smoothed where aberrant pixel values appear.

Obtaining absolute absorption, dark-field and phase modalities requires reference values, which correspond to the value of the modality obtained without sample. These can only be derived in the case of well isolated objects (producing background pixels on the right and left part of each row). In this case an automatic edge detection function is applied defining the reference ‘background’ value for each row. A reference background region can also be manually defined, which is recommended in the case of non-isolated specimens.

In the case of tomographic measurements, sinograms are extracted for each contrast modality from the projection images measured at different angles. For artefact-free tomography reconstruction, parameters such as the position of the axis of rotation have to be known and correction for irregular angular movements (wobble) has to be performed.

*MMX-I* includes correction algorithms for sample wobbling and slight misalignment of the rotation axis. The method, described in detail by Azavedo *et al.* (1990 ▸), consists of computing the centre of mass for each projection and fitting the resulting sinogram curve with a sinus function. In order to perform correctly, this method implies that the object must always be in the field of view of the projections. In addition, special care is given to the sine fit function to minimize the influence of noise. The offset of the fit gives the centre of rotation. The distance between the centre of mass and the fitted curve for each projection is used to correct for the wobble effect. This method requires using preferably a sinogram over 360°. The evaluated shift correction obtained from the modality which has the largest signal contrast is then applied for all the other modalities. In the example shown below, the shift correction is evaluated from the transmission sinograms.

### 2.4. Data reconstruction   

Reconstruction is the last step of data processing in *MMX-I*. In the case of 2D imaging, reconstruction concerns the phase determination from the horizontal and vertical differential phase contrast images. The different phase-retrieval methods implemented in *MMX-I* are presented and compared in the next section.

For tomographic reconstruction, *MMX-I* provides both classical filtered back-projection (FBP) (Kak & Slaney, 2001 ▸) and iterative techniques, such the algebraic reconstruction technique (ART) and the simultaneous iterative reconstruction technique (SIRT) (Kak & Slaney, 2001 ▸). Each tomographic reconstruction method can be optimized independently for each contrast modality in order to extract the correct information. Tomographic reconstruction is described in §2.4.2.

#### 2.4.1. Phase contrast reconstruction   

Two methods have been implemented in *MMX-I* for quantitative phase reconstruction. The first is the Fourier integration method (Kottler *et al.*, 2007 ▸), which applies a Fourier derivative technique to integrate directional phase gradients. The second procedure is the finite-difference-based least-squares integration methods with Southwell (Southwell, 1980 ▸) configuration. The two methods and their performance on quantitative reconstruction of scanning X-ray phase contrast data are described and discussed below.

The Fourier technique is based on the properties of the Fourier transform of a derivative function. A 2D Fourier transform is performed on the complex image formed by the vertical and horizontal phase gradient images as real and imaginary parts, respectively. Thus, the phase image weighted by the complex sum of the frequency coordinates is obtained in the Fourier space. An inverse Fourier transform of the phase image representation in frequency space is performed to determinate the quantitative phase shift φ information in the direct space,
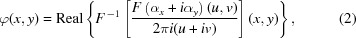
where 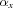 and 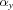 are the horizontal and vertical phase gradients, 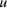 and 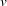 are the reciprocal coordinates, and Real(…) indicates the real part of the complex function. The imaginary part of the inverse Fourier transform contains the error of the phase reconstruction and it is usually represented in terms of intensity less than 10% of the real part (Holzner, 2010 ▸).

**Figure 6:**
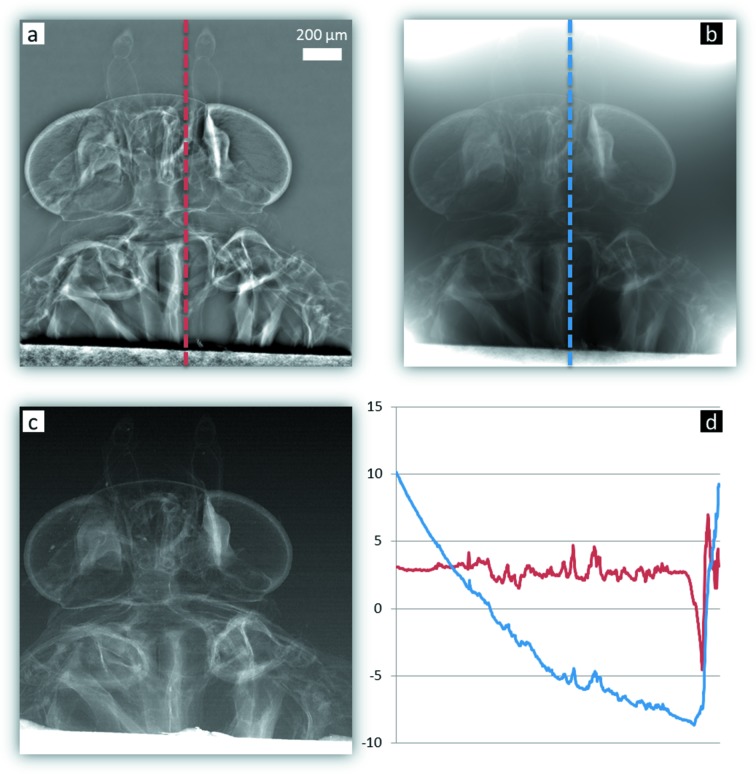
Phase retrieval of a moth head by using Southwell (*a*) and Fourier integration (*b*) techniques. Vertical profiles measured on both images are compared in (*d*). The absorption-contrast image (*c*) shows the high attenuation of the sample holder.

Direct integration using the fast Fourier transform (FFT) algorithm is very fast and easy to implement. The drawback of methods based on discrete Fourier transform (DFT) is that the transformation assumes periodicity in the input image. Hence, when the investigated sample crosses the image boundaries, it will create artefacts in the reconstruction. To overcome this limitation, *MMX-I* offers the user the possibilty to create Von-Neumann boundary conditions by mirror processing in both directions of the differential phase contrast images (see Fig. 6 ▸). The images are mirrored in both directions following either the mirrored derivative integration (MDI) or antisymmetric derivative integration (ASDI) (Bon *et al.*, 2012 ▸). Phase reconstruction is then carried out on the resulting images and automatically cropped to obtain the investigated phase. It has to be noted that, to avoid divergence in equation (2), the zero frequency value of the Fourier transform of the phase is set to zero. This means that the phase offset is lost and the sum of the integrated phase has to be null. Nevertheless, the phase shift can be calibrated using areas of the phase map where no objects are present.

Iterative approaches such as least-squares integration with Southwell configuration methods are well known to be efficient especially when strong discontinuities are present in the DPC images. The Southwell methods consist of the linear integration of the gradients in the horizontal and vertical directions. At each iteration step, the phase shift map 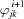 is calculated as follows,
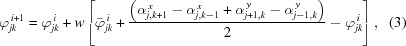
where *i* is the iteration step, *w* the relaxation factor and *j*, *k* the pixel coordinates.

The relaxation factor *w*, which will make the solution converge towards the real phase value, is dependent on the algorithm update strategy. Jacobien and Gaussian Seidel algorithms are proposed to the users. In the first case, the phase is updated when the whole map has been processed. The relaxation factor must be defined in the interval ]0, 1]. In the second case, the phase map is updated after the processing of each pixel. The relaxation *w* has an optimal value, which depends only on *N*, the number of measured gradient values,
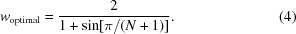
Fig. 6 ▸ presents an example of phase reconstruction, using both integration techniques, performed on scanning imaging data of a head of a moth (Medjoubi *et al.*, 2013*b* ▸). The DPC shows a large discontinuity produced by the strong absorption of the sample holder (Fig. 6*c* ▸). As expected, the Fourier reconstruction technique induced a large low-frequency intensity variation, which contaminates the whole image and restrains any quantitative information. This effect is found to be more localized using the iterative methods: the artefacts are circumscribed in a region around the discontinuity. The figure presents two profiles taken in the phase images reconstructed, respectively, with both methods. It can be seen that the dynamic is strongly reduced with the FFT method compared with the iterative one. Meanwhile, the main drawback of the Southwell technique is the long computation time: it can reach several minutes in the case of a 1 Megapixel projection image like that of the moth.

By proposing in *MMX-I* these two phase integration methods, the user can make a sound compromise between calculation time and the expected more precise quantitative phase reconstruction.

**Figure 7:**
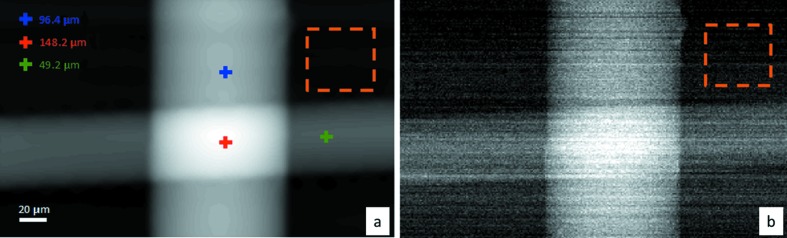
Quantitative thickness reconstruction obtained from phase-retrieval image (*a*) and absorption image (*b*).

Quantitative phase reconstruction has been investigated by using the Fourier integration method. In order to validate the reconstruction performed by *MMX-I*, DPC images of nylon wires have been measured at an energy of 14 keV. The relation among the phase shift φ of the X-ray beam having a wavelength λ within the sample of thickness *t* for which the real part of the refraction index is δ can be written as 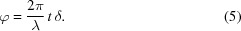
The nominal diameters of the horizontal and vertical wires are 50 µm and 100 µm, respectively. The wire thickness distribution is determined from the phase-shift map by using equation (5) and is shown in Fig. 7(*a*) ▸. The real part of the refraction index at 14 keV and the density of the nylon used are, respectively, 1.63 × 10^−6^ and 1.13 g cm^−3^ (Keyriläinen *et al.*, 2002 ▸). The calculated thickness presents a very good agreement with the nominal values. The error of the thickness reconstruction is estimated to be ∼1 µm RMS from the noise within an area outside of the sample (orange square in Fig. 7*a* ▸). For comparison, the thickness distribution calculated from the absorption image is shown in Fig. 7(*b*) ▸; it is very noisy with a poor signal-to-noise ratio (SNR) compared with the thickness map reconstructed from the phase shift. Moreover, the noise evaluated outside the object is about 12 µm RMS (orange square in Fig. 7*b* ▸).

#### 2.4.2. Multimodal tomographic reconstruction   

X-ray tomographic reconstruction algorithms are well known and well described in the literature (Kak & Slaney, 2001 ▸). Reconstruction algorithms such as FBP (Bracewell & Riddle, 1967 ▸) and ART (Gordon *et al.*, 1970 ▸) are implemented in *MMX-I*. Each processing method has specific filters and reconstruction parameters, which can be optimized for reconstruction calculations of each contrast modality.

FBP is based on the Fourier slice theorem (Kak & Slaney, 2001 ▸). This theorem describes the equality between the Fourier transform of a parallel 1D projection of an object obtained at a given angle and a profile taken at the same angle in the 2D Fourier transform of the object. When including additional projections, the frequency space is filled by additional rotational angles. The over-representation of low frequencies around the zero frequency is corrected by appropriate frequency weighting.

Weight functions such as the ramp function multiplied by a hamming window are widely used as generally they provide the best results for most of the contrast modalities. The differential phase contrast is a specific case. Indeed, by using the Hilbert filter (Pfeiffer *et al.*, 2007 ▸), phase integration is directly obtained from the DPC sinograms. Therefore, these two filters are implemented into *MMX-I*.

FBP is a very fast and robust method. Meanwhile, reconstructed images are artefact-free only if the total number of angular projections is superior or equal to half the mean resolution of the projection (Kak & Slaney, 2001 ▸) and if the SNR is high. In scanning methods, especially for the XRF modality (*e.g.* trace element detection), these two conditions are not always fulfilled. In this case, the iterative method is well adapted. It allows, in spite of being computationally expensive, fewer projections to be handled and low SNR. The method starts with an initial estimate of the density distribution of the object obtained by a simple back-projection. By comparing the projections predicted from this initial estimate with those that are actually acquired, changes are made to the estimated tomogram. Each projection 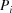 is modelled as follows,
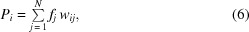
with *i* the projection number (from 1 to *M*, the total number of projections), *j* the pixel number in the tomogram, *N* the total number of pixels of the tomogram image, 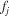 the attenuation value of the pixel *j*, and 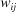 the weighting factor for the *i*th projection and *j*th pixel. This latter corresponds to the fractional area of the *j*th pixel intercepted by the *i*th projection 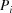. This can also be a logic value (0 or 1) in order to save time for creating the *w* matrix but is less accurate.

Two methods for the determination of 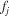 have been integrated into *MMX-I*: standard ART and SIRT (Gilbert, 1972 ▸). In the first case, the 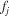 value at the pixel *j* is updated by using one projection at each time. The corresponding expression of 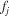 for the *k*th iteration is
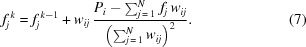
In the SIRT technique, the *f* value at the pixel *j* is updated by using the whole projections. The corresponding expression of *f* for the *k*th iteration is
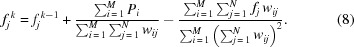
The SIRT technique presents the advantage of strongly reducing the noise and the edge-enhancement artefacts (Leis, 2009 ▸). On the other hand, the SIRT method converges slower and requires more computational resources than the other techniques detailed in this paper.

As previously indicated, iterative approaches (ART and SIRT) are well suited for XRF tomography. In order to illustrate this, XRF tomography datasets acquired on a test sample at an energy of 14 keV (Medjoubi *et al.*, 2013*a* ▸) were processed with *MMX-I*. This test sample consists of a glass capillary of 500 µm diameter containing three nylon fibres (two with a diameter of 100 µm and a 50 µm-diameter one) and a copper wire (40 µm in diameter). A virtual slice of the sample was measured through 500 angular projections equally distributed over 360°. One projection consisted of 500 pixels of 4 µm step size.

**Figure 8:**
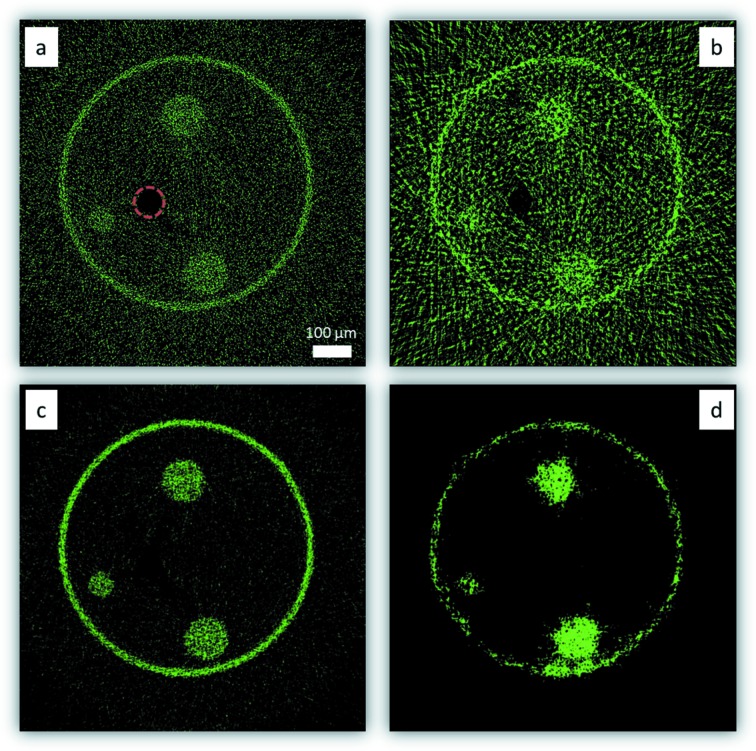
Reconstructed scattering (Rayleigh + Compton) distributions by FBP (top row) and SIRT (bottom row) by using 500 (*a* and *c*) and 30 angular projections (*b* and *d*) of a capillary containing three nylon fibres and a copper wire [marked by the red circle in (*a*) for clarity].

FBP and SIRT tomographic reconstructions have been performed using in one case all the 500 projections and in the other case a sub-dataset of only 30 projections. Results are presented in Fig. 8 ▸.

As expected, tomograms reconstructed with the SIRT method present a better SNR compared with FBP and are presenting fewer artefacts [*e.g.* missing star-like artefacts in Fig. 8(*d*) ▸ compared with Fig. 8(*b*) ▸]. The signal in the XRF sinograms is usually low compared with transmission modalities. Under such conditions, even using the full dataset, the FBP reconstructed tomogram is noisy, as can be seen in Fig. 8(*a*) ▸, which is less the case for the SIRT reconstructed tomogram [*cf.* Figs. 8(*c*) and 8(*d*) ▸]. With a large angular sampling interval, *i.e.* 12° between each projection (30 projections dataset), FBP produces significant star-like artefacts which superimpose on the useful signals. Such artefacts are not observed with SIRT. Moreover, the SNR of the SIRT tomogram is above 5, *i.e.* better than the Rose criteria (Rose, 1948 ▸). The spatial resolution is worse in the case of treating the 30 projections dataset, due to the limited angular projections.

Regardless of the method used, tomographic reconstruction algorithms are based on combining the projections at different angles which requires that the sinograms are corrected for measurement errors, such as those depicted in §2.3, otherwise the reconstruction will contain artefacts resulting in reduced image quality. To treat this problem, correction algorithms have been carefully integrated into *MMX-I*.

### 2.5. User interface   

The graphical user interface (GUI) has been designed in order to guide non-expert users through the complex data processing. It follows the panel structure of the workflow, starting from data importation to phase contrast and tomographic reconstruction. Each panel displays the main information required to validate or tune the default parameters of the actual process. Expert users find more options and parameters by navigating within the dedicated menu bar of each panel. Each step/panel of *MMX-I* is accessible in a specific order, which must be completed sequentially to ensure the input of the essential parameters of data processing. Only when a panel is completed may users advance to the next step, *i.e.* they can proceed to the next panel or they can return to a previous one to tune or add parameters. The GUI is based on the *Swing* platform-independent Java widget toolkit and *ImageJ*, which has been used to display images and provide image interaction with user input.

**Figure 9:**
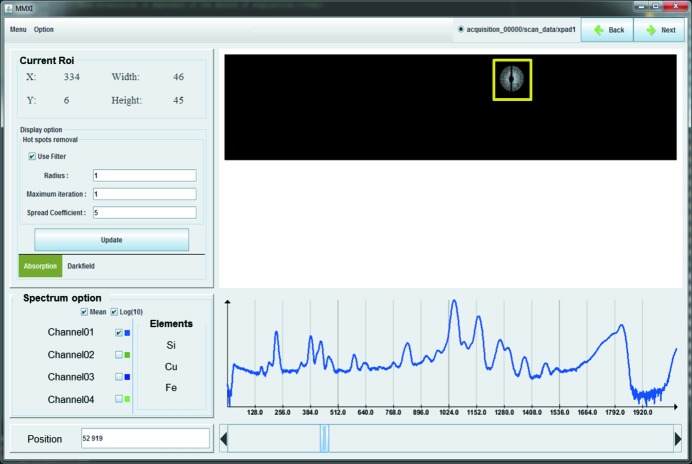
Graphical user interface of *MMX-I*.

The panel of the GUI corresponding to the raw data importation and visualization parts is presented in Fig. 9 ▸. In this panel the raw data of the HDF5 file, *e.g.* measured transmission images (as actually shown in the upper part of the figure), are displayed in the top right of the interface and the XRF spectrum panel in the bottom right. The current ROI for the 2D detector images is automatically shown and can be modified manually by the user. The shown data are updated while navigating with a scroll bar through the measured pixels of the scan. As an option, the sum spectrum of each XRF detector for the whole dataset can also be obtained by selecting the ‘mean function’ as shown in Fig. 9 ▸. This spectrum can be used, for example, for energy calibration. Moreover, ROIs in the XRF spectra, motor position correction and hot pixel removal parameters can be set in this tab.

## 3. Examples   

The possibilities offered by *MMX-I* are illustrated here by some examples. Measurements were performed at the FZP-based micro-probe end-station of Nanoscopium.

### 3.1. Bi-dimensional multimodal imaging of a standard structure   

**Figure 10:**
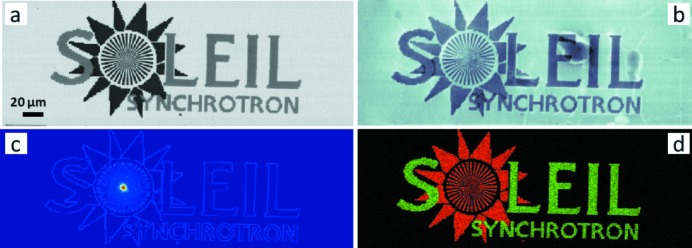
Absorption (*a*), phase (*b*), dark-field (*c*) and XRF (*d*) images of the SOLEIL logo made of gold (in red) and nickel (in green) and with structure size down to 200 nm.

A 2D fast-scanning imaging of a nanostructure calibration chart, which includes the SOLEIL logo (250 µm wide and 75 µm high), was measured with a 400 nm × 400 nm pixel size. For a scan of 300 × 800 pixels the total measurement time was less than 10 min and represents a data volume of 40 GBytes. The absorption contrast, differential phase contrast, dark-field contrast, phase contrast and Au, Ni maps were reconstructed with *MMX-I* in less than 5 min with a laptop (Intel core i7-4900MQ, 16Go Ram, 1To Samsung SSD EVO 840) and can be seen in Fig. 10 ▸. The reconstructed phase shifts of the gold and nickel structures are 0.6 and 0.66 rad, respectively, which agree well with the thickness values extracted from the absorption image.

### 3.2. Multitechnique tomography of a microfossil   

Microfossils are valuable tools for paleo-environmental interpretations. Due to their ubiquity in most marine environments and their abundance in the geological records from the Cambrian (>500 million of years) to the present times, foraminifera are one of the most widely studied groups of fossil records worldwide in paleoceanography (Fischer & Wefer, 1999 ▸).

Foraminifera are single-cell eukaryotes (Protozoa). Many species secrete shells, which are composed of calcium carbonate (CaCO_3_). During its secretion, trace elements are incorporated into this calcareous shell from the sea water (Boyle, 1981 ▸; Munsel *et al.*, 2010 ▸), in concentrations depending on the ambient sea water conditions. As such, geochemical data obtained from foraminifera measurements are used as proxies also in paleo-climatology. Moreover, if we can understand how organisms in the past responded to environmental changes, then that information can be used to predict how future natural or anthropogenic environmental change might affect the Earth’s biota. Understanding mechanisms of heavy metals incorporation during biomineralization processes in foraminifera is a fundamental key for interpretation of these geological records, where spatially resolved and high analytical sensitivity 3D elemental and morphological studies might bring important new information to the existing mostly bulk studies.

**Figure 11:**
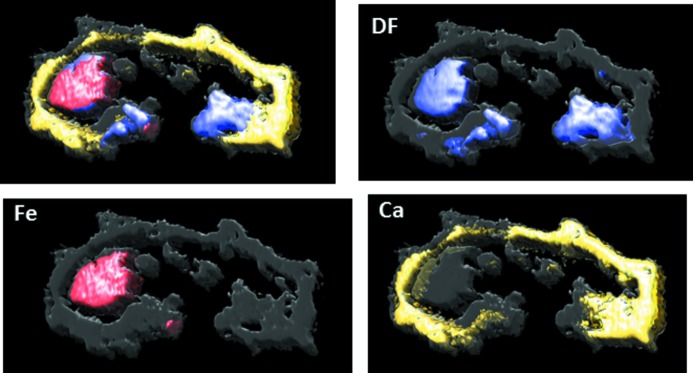
Multimodal volume rendering of a foraminifera sample. The reconstructed phase is shown in grey.

In order to test the possibility of scanning hard X-ray tomography on such samples, we performed 3D multi-technique tomography on a fossil foraminifera. Tomography has been performed with an angular step 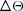 = 1.8° over 180°. We aimed at the acquisition of moderate 2 µm × 2 µm spatial resolutions, due to the large 300 µm × 100 µm × 120 µm size of the sample. We measured 23 virtual slices in 1 µm steps between the slices. The acquisition was accomplished slice by slice in the *x* (scanning line), 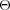 (rotation in the axis perpendicular to the scanning line) and *z* (axis perpendicular to the scanning line) sequence, by scanning a full horizontal line with 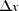 = 1 µm steps and then performing a rotational step (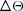). The measurement time was 3 ms per pixel. The total measurement time of the 500 × 200 × 23 voxels, each containing the XRF, the transmission data, data coordinate *etc.* as described above, was 3 h. It resulted in a 500 GB data file. This dataset was analysed and the tomograms of the different modalities were reconstructed by *MMX-I*. In Fig. 11 ▸ the volume rendering of the reconstructed phase, dark field and the distributions of Fe and Ca are shown. The best morphological contrast is obtained by DPC in the case of the foraminifera, which contains a thin (∼10 µm) CaCO_3_ shell. The highest dark-field contrast indicating highly scattering granular structure corresponds to structures with lower absorption, which contain no Ca. These light highly scattering structures can be found mostly in the interior of the shell. Interestingly, Fe is distributed within the highly scattering internal structure and also as a small Fe grain at the exterior of the foraminifera. No significant Fe was found within other highly scattering structures.

## 4. Discussion and conclusion   

The *MMX-I* software developed at the Nanoscopium beamline of SOLEIL is, to our knowledge, the first software tool that aims at treating all modalities (except ptychography) of scanning multi-technique imaging and tomography experiments.

*MMX-I* includes different data-correction and reconstruction methods both for 2D imaging and tomography. For each included contrast modality, which up to now is scanning XRF, differential phase contrast, absorption contrast and dark field, the software offers a dedicated default data-treatment and tomographic reconstruction method which is, according to our experience, the best suited to a given modality for most sample types. As such, users unfamiliar with X-ray imaging can readily use the software. This is well illustrated by the examples of the foraminifera and the standard structure, where the default ROI, ‘illumination mask’, reconstruction methods and variables were used for data treatment. Users experienced in the treatment of X-ray imaging have the possibility to modify and optimize all the parameters. This diversity and flexibility makes *MMX-I* a software tool well adapted to data treatment of fast multimodal imaging. Multimodal imaging opens the additional possibility of the combination of all modalities in order to obtain complete information for quantification. For light elements in strongly absorbing sample matrices, self-absorption correction of XRF tomographic data is crucial, which will be developed as a further option in *MMX-I*.

The modular construction of *MMX-I* makes the integration of other imaging modalities or reconstruction algorithms (Gürsoy *et al.*, 2015 ▸) easy and readily available. For example, it is possible to integrate other methods, *e.g.* full-field tomography, as a new modality which could be useful for correlative imaging. However, *MMX-I* does not intend to replace the already existing powerful full-field tomographic reconstruction software tools.

In addition, one of the important features of the software is the dedicated data read-in API, named Hdf5Opener, which makes the read-in and treatment of large, several hundred GB, data files possible also with standard and laptop user PCs. This is a crucial criterion for its utilization by a large user community. Moreover, fast data read-in provided by the beamline data-treatment server makes data pre-treatment and tomographic reconstruction possible during user experiments. The standalone Hdf5Opener API can be readily used by other software.

The *MMX-I* freeware is available for data treatment of scanning multimodal imaging both for users at the Nanoscopium beamline and for other user communities. The compiled version of *MMX-I* is available at https://bitbucket.org/antoinebergamaschi/mmx-i/wiki/Home. The source code is available from the authors on demand.
